# Distribution of Parkinson’s disease associated RAB39B in mouse brain tissue

**DOI:** 10.1186/s13041-020-00584-7

**Published:** 2020-03-30

**Authors:** Yujing Gao, Gabrielle R. Wilson, Sarah E. M. Stephenson, Mustapha Oulad-Abdelghani, Nicolas Charlet-Berguerand, Kiymet Bozaoglu, Catriona A. McLean, Paul Q. Thomas, David I. Finkelstein, Paul J. Lockhart

**Affiliations:** 1 Bruce Lefroy Centre for Genetic Health Research, Murdoch Children’s Research Institute, 50 Flemington Road, Parkville, Victoria 3052 Australia; 2 Department of Paediatrics, The University of Melbourne, 30 Royal Parade, Parkville, Victoria 3052 Australia; 3 Institut de Génétique et de Biologie Moléculaire et Cellulaire (IGBMC), INSERM U964, CNRS UMR7104, Strasbourg University, 67400 Illkirch, France; 4 Anatomical Pathology, Alfred Hospital, Melbourne, Victoria 3004 Australia; 5 Robinson Research Institute and School of Medicine, University of Adelaide, Adelaide, SA 5005 Australia; 6 The Florey Institute of Neuroscience and Mental Health, The University of Melbourne, 30 Royal Parade, Parkville, Victoria 3052 Australia

**Keywords:** RAB39B, RAB GTPase, Protein localization, Parkinsonism, Parkinson’s disease, Mouse model, Knockout mouse

## Abstract

Pathogenic variants in the gene encoding the small GTPase Ras analogue in Brain 39b (RAB39B) are associated with early-onset parkinsonism. In this study we investigated the expression and localization of RAB39B (RNA and protein) in mouse brain tissue to gain a better understanding of its normal physiological function(s) and role in disease.

We developed novel resources, including monoclonal antibodies directed against RAB39B and mice with *Rab39b* knockout, and performed real-time PCR and western blot analysis on whole brain lysates. To determine the spatial localization of Rab39b RNA and protein, we performed in-situ hybridization and immunohistochemistry on fresh frozen and fixed brain tissue. Our results show that RAB39B is localized throughout the cortex, hippocampus and substantia nigra of mice throughout postnatal life. We found high levels of RAB39B within MAP2 positive cortical and hippocampal neurons, and TH positive dopaminergic neurons in the substantia nigra *pars compacta*.

Our studies support and extend current knowledge of the localization of RAB39B. We validate RAB39B as a neuron-enriched protein and demonstrate that it is present throughout the mouse cortex and hippocampus. Further, we observe high levels in the substantia nigra *pars compacta*, the brain region most affected in Parkinson’s disease pathology. The distribution of Rab39b is consistent with human disease associations with parkinsonism and cognitive impairment. We also describe and validate novel resources, including monoclonal antibodies directed against RAB39B and mice with *Rab39b* knockout, both of which are valuable tools for future studies of the molecular function of RAB39B.

## Introduction

Parkinson’s disease (PD) is a prevalent neurodegenerative disorder characterized by the manifestation of motor deficits, and neuropathological features including neuron loss and alpha-synuclein (αSN) aggregates in the substantia nigra *pars compacta* (SNpc). Currently, the majority of PD cases are idiopathic, although in ~ 10–15% of cases there is an underlying genetic cause [1]. The study of genetic forms of PD has implicated disrupted cellular trafficking, mitochondrial function and protein degradation in the pathological mechanisms underlying PD. Thus, the discovery of novel genetic forms of PD remain integral to the field, as it improves our understanding of the interplay between dysregulated pathways and mechanisms contributing to the disease and identifies potential therapeutic targets.

Loss of function variants in the gene encoding the small GTPase protein, Ras Analogue in Brain 39b (*RAB39B*) were recently shown to cause an X-linked recessive form of early-onset parkinsonism [2]. RAB39B is a neuron enriched GTPase belonging to the RAB superfamily of proteins. In general, these proteins function to regulate a range of intracellular trafficking events by cycling between GDP-bound inactive and GTP-bound active forms. Previous reports have implicated RAB39B in the regulation of αSN homeostasis, synapse formation, neurotransmitter release and autophagy pathways [2–7]. Further, RAB39B was identified as a potential in vitro substrate for another PD-associated protein Leucine-rich repeat kinase 2 (LRRK2) [8]. However, the relevance of the potential functional interactions between RAB39B and αSN or LRRK2 in vivo, and the contribution of its putative functions to the pathogenesis of PD remains unknown and requires further investigation.

The functional specificity of RAB GTPases can be determined by their localization and the identity of their interactor proteins. Determining the localization of RAB *GTPases* is an important step towards identifying potential interactors and/or cargo that the protein can regulate. Currently, one study has investigated the expression and localization of *Rab39b* in brain tissue [3]. The study demonstrated that *Rab39b* was enriched in human and mouse brain tissue. Bulk analysis of mouse brain tissue at postnatal day one to 180 by real-time PCR showed that *Rab39b* expression was predominantly occurring in neuronal precursors and neurons, as opposed to microglia and oligodendrocytes. A significant upregulation of expression was identified between postnatal day 10 and 20. Further, in-situ hybridization suggested the highest expression was in the hippocampus [3]. Correspondingly, a second study reported localization of RAB39B to mossy fiber synapses of the hippocampus by immunohistochemistry [9]. However, localization of RAB39B to other brain regions was not explored in this study and remains largely unknown.

To address this knowledge gap, we generated and validated monoclonal antibodies for RAB39B and mouse models with knockout (KO) of *Rab39b* and investigated the spatiotemporal localization of RAB39B (RNA and protein) in mouse brain tissue.

## Materials and methods

### Generation of Rab39b knockout mice

Mice were housed in a specific-pathogen free environment under a 12 h light/dark cycle with free access to food and water. *Rab39b* KO mice were generated using CRISPR/Cas9 by the South Australian Genome Editing Facility (Adelaide, Australia). In brief, CRISPR guides flanking exon one of *Rab39b* (supplementary Table 1) were injected into c57Bl/6 J mouse zygotes, and the resulting genotypes confirmed by Sanger sequencing of genomic DNA extracted from tail biopsies (supplementary Table 2). Potential Cas9 off-target sites were predicted using the CRISPR/Cas9 target online predictor (CCTop). Colonies were established by backcross of hemizygous KO male mice to the wildtype females for a minimum of five generations prior to tissue collection and data analysis.

### RAB39B recombinant protein production, purification and antibody development

*E. coli* BL21(RIL) pRARE competent cells (Invitrogen) were transformed with pET28a-GST_RAB39B and grown at 30 °C to an OD600 of 0.5. Recombinant protein expression was induced with 0.5 mM IPTG for 4 h and GST-tagged proteins were purified using the GST-Bind™ Kit (Novagen). For generation of mouse monoclonal antibodies, 100 μg of purified GST-RAB39B and 200 μg of poly (I/C) adjuvant were injected intraperitoneally into 2 months old BALB/c female mice. Three injections were performed at 2 week intervals. Spleen cells were fused with Sp2/0.Agl4 myeloma cells and hybridoma culture supernatants were tested by ELISA for reaction with GST-RAB39B. Specific cultures were cloned twice on soft agar. Two specific clones, 1H1 and 1B8, were established and ascites fluid was prepared by injection of 2 × 10^6^ hybridoma cells into Freund adjuvant-primed BALB/c mice. All animal experimental procedures were performed according to the European authority guidelines.

### RNA extraction and PCR

Total RNA was extracted from brain tissue using the SV Total RNA Isolation System (Promega) according to the manufacturer’s protocol and RNA integrity was evaluated using Nanodrop ND-2000 (Thermo Scientific). cDNA was synthesized from one μg of total RNA using the Transcriptor First Strand cDNA Synthesis Kit (Roche). PCR analysis of cDNA was performed using MyTaq DNA Polymerase (Bioline) with a T100 Thermal Cycler (Biorad) (supplementary Table 2). Amplified products were resolved by gel electrophoresis in 1% Agarose (Bioline). Quantitative real time PCR (qRT-PCR) analysis was performed with a LightCycler LC480 II (Roche) and taqman probes (Life Technologies) specific for mouse *Rab39b* (Mm00838519_m1) and *Tbp* (Mm00446971_m1). Relative expression was calculated using the Pfaffl ddCT method [10].

### Protein extraction and western blotting

Western blot analysis was performed as previously described [11]. Briefly, total protein was extracted from brain tissue using a buffer containing 2% SDS and 1x Protease inhibitor (Sigma). Lysates were sonicated and total protein concentration estimated by Bicinchoninic acid assay (Pierce). For micro-dissections, whole brains were sliced into 2 mm coronal sections using a mouse brain slicer matrix (Zivic), then further dissected to isolate specific brain regions under a stereomicroscope (Leica). Western blot analysis was performed using 20 μg of total protein separated on 12% SDS-PAGE and transferred onto 0.45 μm-pore PVDF membranes (Immobilon-P) at 10 V overnight. Following transfer, membranes were blocked in 5% skim milk for 2 h, then incubated with primary antibodies at 4 °C overnight. Antibody binding was revealed using horseradish peroxidase-conjugated secondary antibodies (Jackson Laboratories) and enhanced chemiluminescence (Bio-Rad) according to manufacturer’s protocol. Images were captured with an ImageQuant LAS4000 and quantified using ImageQuantTL software (GE Healthcare).

### In-situ hybridization (ISH)

ISH was performed essentially as previously described [12]. Briefly, mice were culled by cervical dislocation and whole brains were immediately dissected, fixed in 4% paraformaldehyde (PFA) overnight, cryoprotected in 20% sucrose overnight at 4 °C, and then snap frozen in iso-pentane over dry-ice. Serial sections of 10 μm thickness were cut and collected on superfrost adhesion slides (Menzel-Glaser). To generate sense and anti-sense ssRNA riboprobes, the target *Rab39b* exonic sequences were amplified using specific primers and cloned into PCRII TOPO plasmid (Invitrogen) (supplementary Table 3). Riboprobes were Digoxigenin (DIG) labelled using the DIG RNA labelling kit (Roche) according to manufacturer’s instructions and hybridized to tissue sections at 64 °C in a sealed chamber overnight. Probe binding was detected by incubation with an alkaline phosphatase conjugated anti-DIG antibody (Roche, 11,093,274,910) and visualized through NBT/BCIP reaction (Roche). Slides were mounted with aqueous mounting agent (Merck) and viewed and imaged with an optical microscope (Olympus).

### Immunohistochemistry (IHC)

Mice were culled by cervical dislocation and whole brains were immediately dissected, fixed in 4% PFA overnight, then cryoprotected in 30% sucrose for up to 1 week at 4 °C before sectioning. Serial sections of 10 μm thickness were collected on superfrost adhesion slides (Menzel-Glaser). Slides were blocked and permeabilized in 0.2% Triton™ X-100 (Sigma), 3% Bovine serum albumin (Sigma) and 10% Goat serum (Life Technologies) for 1 h, incubated with primary antibodies for 48 h at 4 °C, then secondary antibodies (AlexaFluor 488 or 594, Invitrogen) for 1 h. Slides were mounted with Vectashield containing DAPI (VectorLabs) and analyzed with a confocal microscope (LSM 780, Carl-Zeiss) using ZEN black image processing software. Primary antibodies utilized include rabbit anti-RAB39B (Proteintech, 12,162–1-AP), sheep anti-tyrosine hydroxylase (TH) (Merck Millipore, ab1542), and mouse anti-microtubule-associated protein 2 (MAP2) (Sigma Aldrich, M1406).

### Statistical analysis

Statistical significance was determined using unpaired student t-tests (Graphpad Prism 7, CA, USA). All quantified data are displayed as mean +/− standard error of the mean (SEM).

## Results

### Development of Rab39b knockout mice

**Fig. 1:**
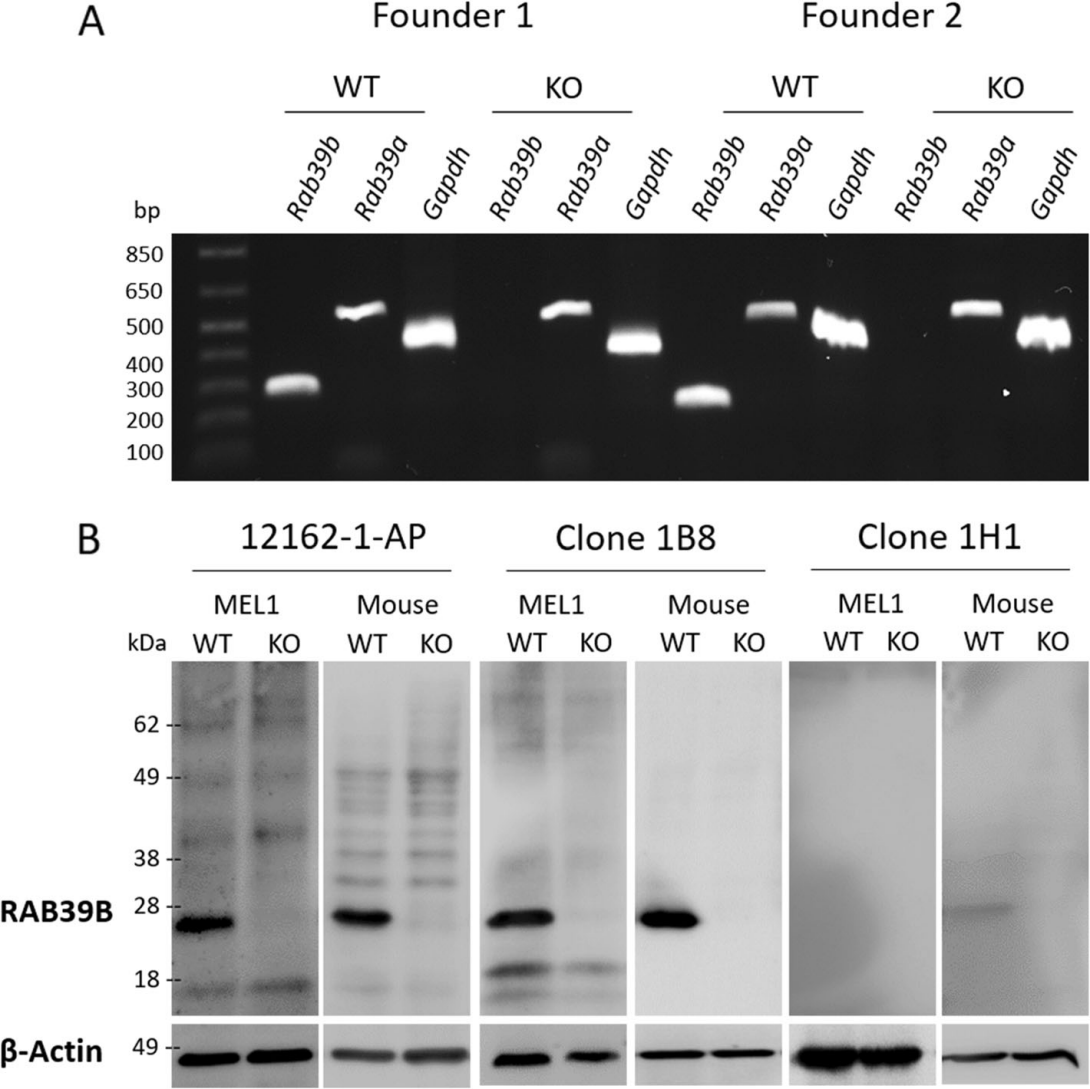
Validation of *Rab39b* KO mice and monoclonal antibodies. **a** PCR analysis of *Rab39b* expression in brain tissue from two independent founder strains confirms complete KO of *Rab39b* and retained expression of *Rab39a* and *Gapdh*. **b** Evaluation of the specificity of RAB39B antibodies 12,162–1-AP (Proteintec), 1B8 and 1H1 by western blot analysis of human control and RAB39B KO tissue (derived from embryonic stem cell line MEL-1) and mouse wildtype and Rab39b KO tissue

*Rab39b* KO mice were established via CRISPR/Cas9 genome editing. We utilized CRISPR guides targeting the intronic sequences flanking *Rab39b* exon one with minimal off-target sites predicted (supplementary Table 1), and injected c57Bl/6 J mouse zygotes. Cas9-mediated cleavage of DNA followed by non-homologous end joining repair resulted in mice carrying partial deletions of *Rab39b* exon one. Seven *Rab39b* KO founder mice were generated, comprising one male and six female mice. Sequencing analysis of genomic DNA identified two independent founders with deletions spanning the initiating methionine and more than 70 N-terminal amino acids of the 214 amino acid protein. Specifically, one founder possessed a 259 base pair deletion (NM_175122.6:c.-24_235del), and the second founder a 234 base pair deletion (NM_175122.6:c.-4_230del). Analysis of brain tissue derived cDNA by PCR (supplementary Table 2) showed complete loss of *Rab39b* in mice derived from the two founders, and retained expression of the homologous *Rab39a*; integrity of the cDNA was confirmed by analysis of the *Gapdh* (Fig. 1a).

### Development and evaluation of monoclonal RAB39B antibodies

We generated two mouse monoclonal antibodies directed against RAB39B (clone 1B8 and 1H1) and tested their specificity and sensitivity by western blot analysis. These novel reagents were compared to a commercially available and previously validated RAB39B antibody (12162–1-AP, Proteintech) [2]. We utilized extracts from MEL-1 human embryonic stem cell line with CRISPR/Cas9 KO of *RAB39B* (MEL-1 30.3) [13] and *Rab39b* KO mice, along with corresponding unmodified parental lines. Clone 1B8 showed the greatest sensitivity and specificity for RAB39B, with no signals detected in KO lysates (Fig. 1b). All subsequent western blot analyses were performed using this antibody.

### Spatiotemporal localization of RAB39B in mouse brain tissue

**Fig. 2:**
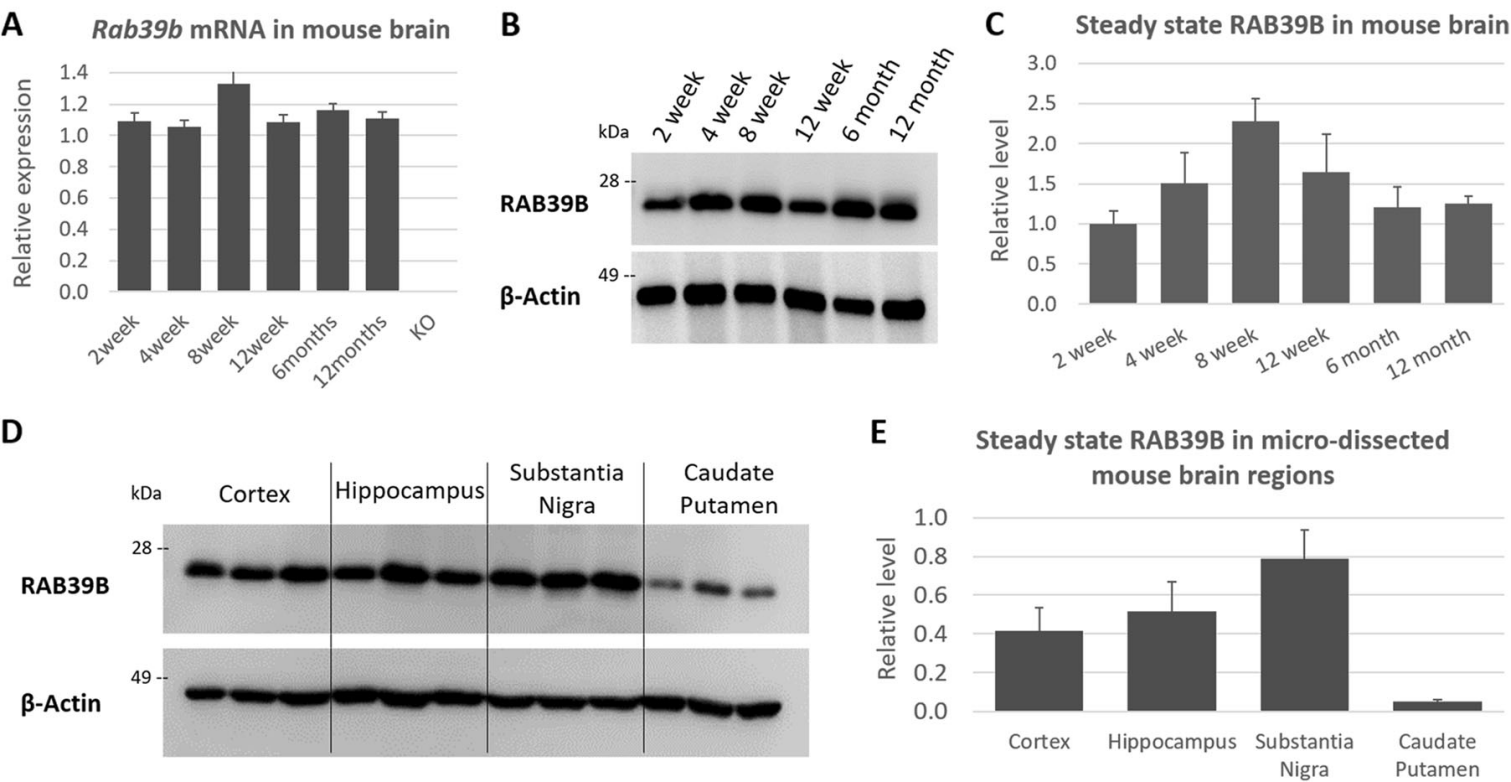
Analysis of RAB39B by RT-PCR and western blot. **a** Quantification of *Rab39b* expression relative to *Tbp* in whole mouse brain from 2 weeks to 12 month of age (mean ± SEM, *n* = 3 per time point). **b** Representative western blot of steady state RAB39B in whole mouse brain from 2 weeks to 12 month of age. **c** Quantification of RAB39B steady state levels relative to β-Actin in whole mouse brain from 2 weeks to 12 month of age (mean ± SEM, *n* = 3 per time point). **d** Representative western blot of steady state RAB39B in micro-dissected mouse brain (*n* = 3 per tissue region). **e** Quantification of RAB39B steady state levels relative to β-Actin in micro-dissected mouse brain (mean ± SEM, *n* = 3)

To determine the temporal localization of RAB39B (RNA and protein), we performed qRT-PCR and western blot analysis using mouse whole brain extracts. We demonstrated consistent expression of *Rab39b* (Fig. 2a) and steady state levels of the protein (Fig. 2b, c) from 2 weeks to 12 month of age, suggestive of stable expression throughout postnatal life. We also assessed RAB39B levels in micro-dissected brain regions by western blot analysis. We analyzed regions that were shown to possess high levels of gene expression previously, the cortex and hippocampus, in addition to the substantia nigra (SN) and caudate putamen (CPu); regions associated with pathology in human parkinsonism. Relative quantification of RAB39B levels showed moderate to high levels of steady state RAB39B in the cortex, hippocampus and SN, and potentially low levels in the CPu (Fig. 2d, e).

**Fig. 3:**
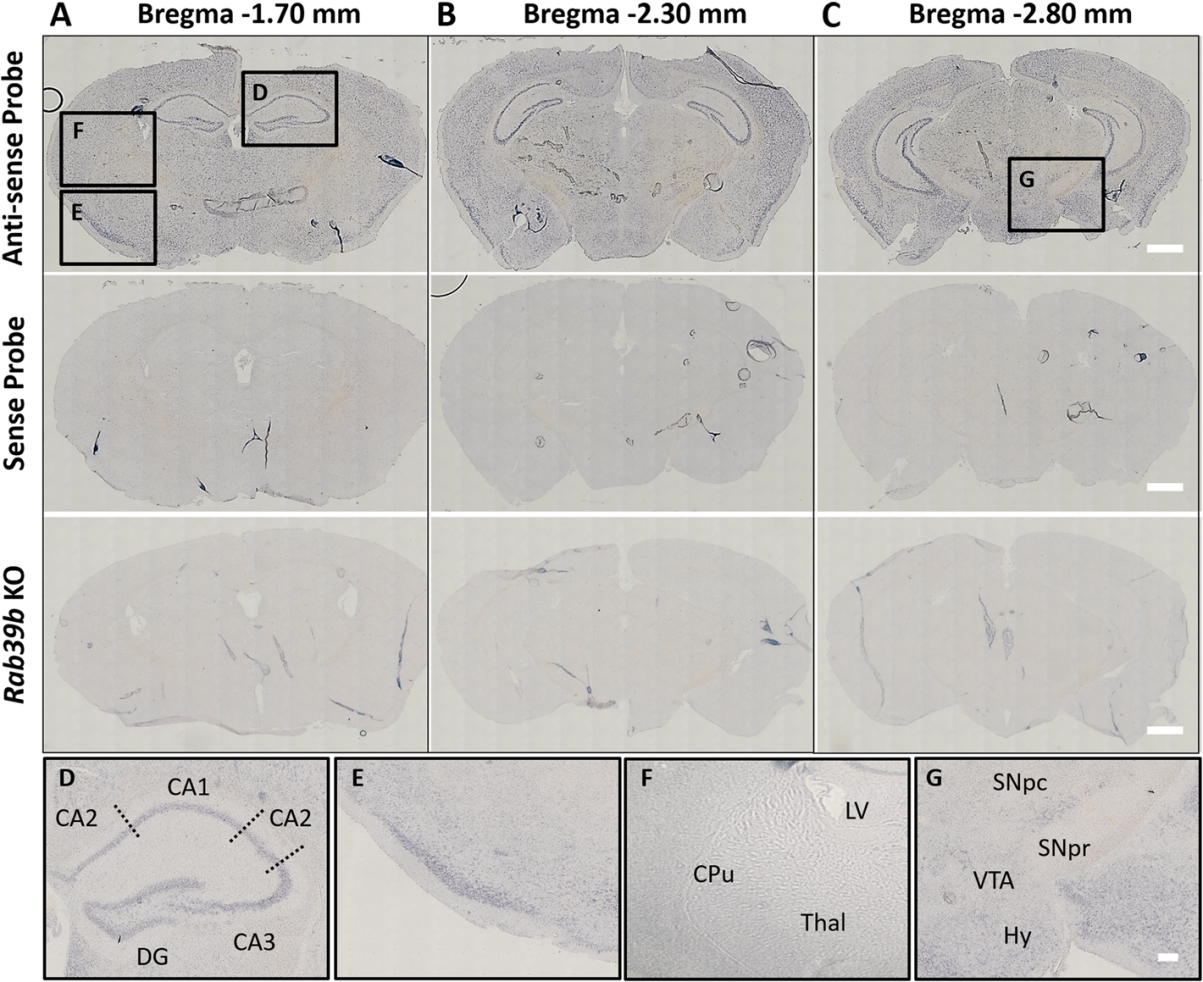
Distribution of *Rab39b* by ISH in mouse brain tissue. Representative images of *Rab39b* localization by ISH in coronal sections of 1 month old mouse brain (*n* = 3) at Bregma coordinates (**a**) -1.70 mm, (**b**) -2.30 mm and (**c**) -2.80 mm. Scale bar = 500 μm. (**d**-**g**) High magnification images of *Rab39b* localization by ISH at the (**d**) hippocampus, (**e**) cortex, (**f**) caudate putamen and (**g**) SN. Scale bar = 100 μm

To investigate the regional distribution of *Rab39b* expression, we performed ISH with two independent RNA probes on fresh frozen and fixed brain tissue of 1 month old mice (Fig. 3). For all sections analyzed, the specificity of probe staining was confirmed by comparison to *Rab39b* KO tissue and the sense probe. We show that *Rab39b* is abundantly expressed throughout the cortex and hippocampus. In the hippocampus we found the highest expression to be in the pyramidal cells of the CA1–3 regions, and the granule cell layer of the dentate gyrus (Fig. 3d). We observed expression throughout cortical layers, in particularly at the piriform cortex (Fig. 3e). Relatively low expression was observed within the nigrostriatal pathway, including the CPu and thalamus (Fig. 3f), with the notable exception of the SN. Significant expression was evident in the SN *pars compacta* (SNpc) as opposed to the SN *pars reticulata* (SNpr) (Fig. 3g).

**Fig. 4:**
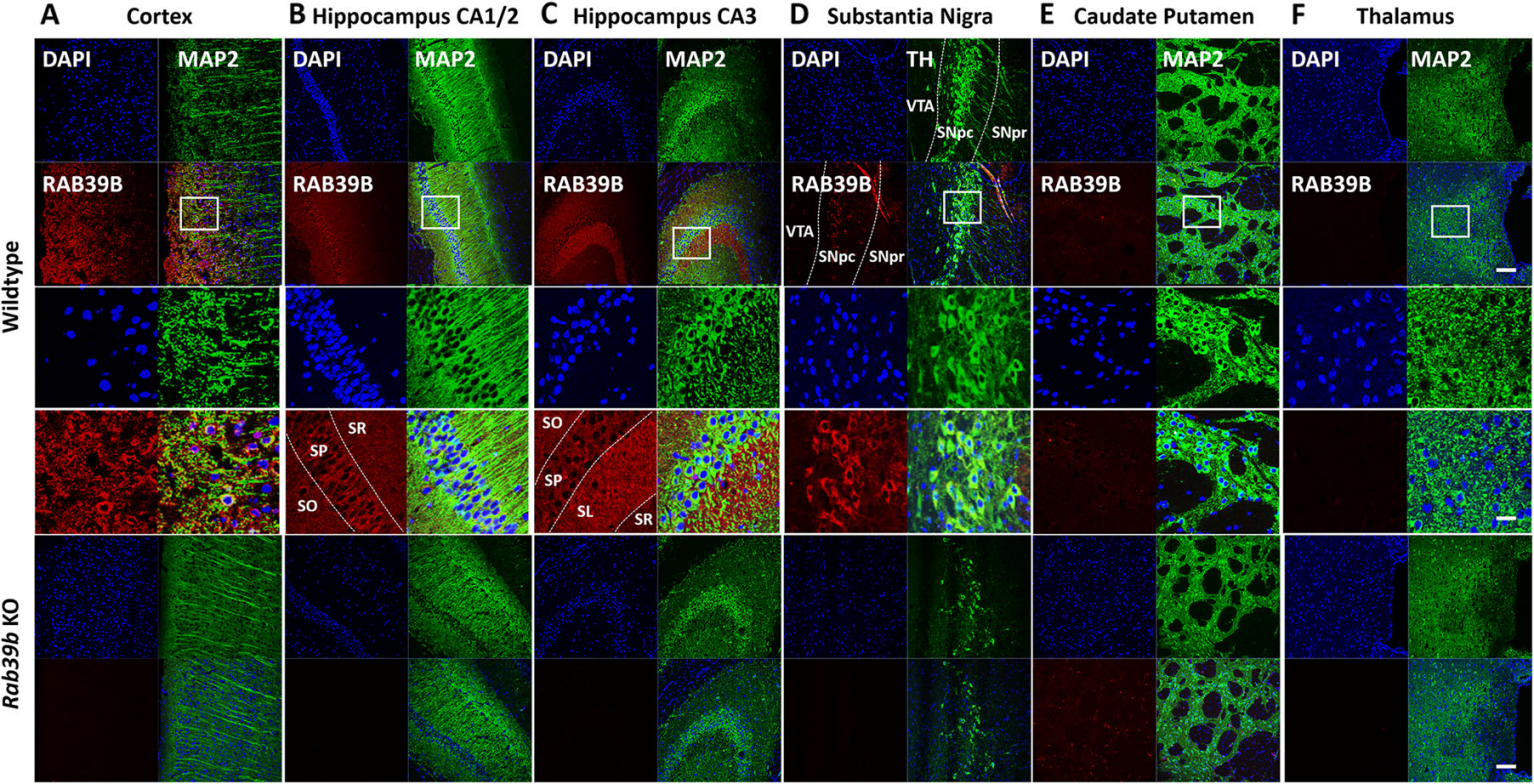
Distribution of RAB39B by IHC in mouse brain tissue. Representative images of RAB39B localization by IHC in coronal sections of 1 month old mouse brain (*n* = 3) at the **a** cerebral cortex co-stained with MAP2, **b**, **c** hippocampus co-stained with MAP2, **d** SN co-stained with TH, **e** caudate putamen co-stained with MAP2 and **f** thalamus co-stained with MAP2. Boxed areas in upper panels (scale bar = 200 μm) are shown at a higher magnification in the corresponding lower panels (scale bar = 50 μm)

To investigate the distribution of RAB39B protein, we performed IHC analysis on fresh frozen and fixed brain tissue collected from 1 month old mice (Fig. 4). For all sections analyzed, the specificity of antibody staining was confirmed by comparison to *Rab39b* KO tissue and staining observed when using only the secondary antibody. We observed RAB39B in the soma of neurons throughout the cerebral cortex (Fig. 4a) and hippocampus (Fig. 4b, c), co-localizing with the pan-neuronal marker MAP2. Strong signal was observed in the hippocampus, in particular the pyramidal neurons of CA1–3 regions (Fig. 4b), and also in mossy fibers of the CA3 region (Fig. 4c). Further, high levels of immunoreactivity for RAB39B was observed in the soma of neurons throughout the SNpc, co-localizing with the dopaminergic neuron marker TH (Fig. 4d). In contrast, there was minimal staining in the SNpr or the ventral tegmental area (VTA). We did not detect RAB39B in the CPu (Fig. 4e) and thalamus (Fig. 4f) by IHC.

## Discussion

*RAB39B* is a novel PD associated gene with a putative role in intracellular trafficking. Currently, little is known about the distribution of RAB39B in brain tissue, and how dysregulation of its function(s) can lead to the development of disease. To address this knowledge gap, we generated mouse models with KO of *Rab39b*, and investigated the distribution of RAB39B (RNA and protein) in mouse brain tissue. In this study, we showed that RAB39B is present throughout postnatal life, with the protein being abundant in the neuronal soma of cortical and hippocampus, which have implications for its proposed roles in synaptic maintenance and cognition. These results are consistent with and extend previous reports examining the localization of *Rab39b* expression and RAB39B protein in mouse brain tissue [3, 9]. Notably, we demonstrated that RAB39B is an abundant protein in dopaminergic neurons in the SNpc, the neuronal subtype selectively lost in PD.

The distribution of RAB39B (RNA and protein) in cortical and midbrain regions could potentially explain the wide spectrum of clinical features that have been observed in individuals with mutations in *RAB39B*. Specifically, the function(s) of RAB39B in cortical and hippocampal regions are likely to be associated with the development of cognitive impairment, whilst its function(s) in the SNpc could be linked to the development of parkinsonism. Consistent with the mouse data reported herein, we have recently demonstrated substantial steady-state levels of RAB39B in human cortex, hippocampus and substantia nigra [14]. These observations suggest the mouse represents an appropriate model for further studies to investigate the pathological mechanisms underlying parkinsonism and cognitive dysfunction in *RAB39B*-mediated disease. Whilst the role of RAB39B in cognitive function is relatively well established, a specific role in the pathogenesis of parkinsonism is yet to be elucidated. Therefore, additional investigations into the distribution of RAB39B in other human brain regions affected in parkinsonism beyond the SN and CPu could help define its role in disease mechanisms.

The abundance of RAB39B protein in neuronal somas is suggestive of a role for RAB39B in trafficking events in the cell body as opposed to axonal projections and neuronal synapses. This hypothesis is supported by the lack of RAB39B localized to dopaminergic synapses in the striatum by ISH and IHC. To date, the intracellular localization of endogenous RAB39B has not been well defined in vitro or in vivo. However, one study investigated the intracellular localization of RAB39B using neural differentiated PC12 and SK-N-BE [2] C cells overexpressing *RAB39B*. Immunofluorescent microscopy showed that wildtype RAB39B could be observed throughout the cytoplasm of cell bodies, at the plasma membranes and at neuritic processes [15]. In order to definitively define the specific intracellular localization of endogenous RAB39B, further studies are required, which will be facilitated by the mouse models generated in this study, in addition to previously generated stem cell models [13].

In conclusion, we have expanded on current knowledge of the distribution of RAB39B in mouse brain, and demonstrated that neuronal localization of the endogenous protein is not limited to cortical regions as has been previously reported. Further, we generated and validated monoclonal antibodies for RAB39B and mouse models with KO of *Rab39b*. Ongoing studies are now investigating the subcellular localization of RAB39B and testing the behavioral and biochemical phenotypes observed in aged KO mice. These novel resources represent valuable tools for future investigations into the physiological function(s) of RAB39B and its role in human disease states.

## Supplementary information

**Additional file 1: Table S1.** Predicted CRISPR/Cas9 targets and off- targets. **Table S2.** Genotyping primers. **Table S3.** Riboprobe primers.

**Additional file 2: Figure 1.** Control experiments for IHC.

## Data Availability

All data generated or analysed during this study are included in this published article.

## Abbreviations

PD: Parkinson’s disease
RAB39B: Ras Analogue in Brain 39b
αSN: α-synuclein
LRRK2: Leucine-rich repeat kinase 2
TH: Tyrosine Hydroxylase
MAP2: Microtubule-associated protein 2
qRT-PCR: quantitative real time PCR
ISH: in-situ hybridisation
IHC: Immunohistochemistry
WT: Wildtype
KO: Knockout
SN: Substantia nigra
SNpc: Substantia nigra *pars compacta*
SNpr: Substantia nigra *pars reticulata*
VTA: Ventral tegmental area
Hy: Hypothalamus
CPu: Caudate putamen
LV: Lateral ventricle
Thal: Thalamus
DG: Dentate gyrus
SO: Stratum oriens
SP: Stratum pyramidale
SL: Stratum lucidum
SR: Stratum radiatum
