# Supplementary material for: Distribution of Parkinson’s disease associated RAB39B in mouse brain tissue
Source: Mol Brain. 2020 Mar 30;13:52. doi: 10.1186/s13041-020-00584-7 (PMC7106796; doi:10.1186/s13041-020-00584-7)
Supplement: Supplementary file 1 — Additional file 1: Table S1. Predicted CRISPR/Cas9 targets and off- targets. Table S2. Genotyping primers. Table S3. Riboprobe primers. [file 13041_2020_584_MOESM1_ESM.docx]

**Supplementary data**

**Table S1** Predicted CRISPR/Cas9 targets and off-targets (Top 20)

| **Guide 1** | **GTACAGCCAGATGGCCTCCATGG** | | | | |
| --- | --- | --- | --- | --- | --- |
| **target_seq** | **mismatches** | **locus** | | **gene** | |
| GTACAGCC[AGATGGCCTCCA] | 0 | chrX exonic | | *Rab39b* | |
| GCTTAGGC[AGATGGCCTCCA] | 4 | chr10 intronic | | *Unc5b* | |
| CAACAGTC[TGATGGCCTCCA] | 4 | chr10 intergenic | | *NA* | |
| TTACTGGA[AGATGGCCTCCA] | 4 | chr3 intergenic | | *Gm38030* | |
| CGACATCC[ACATGGCCTCCA] | 4 | chr14 intergenic | | *Slc7a8* | |
| ATTCAACC[AGCTGGCCTCCA] | 4 | chr6 intronic | | *Gm22971* | |
| CTACATAC[AAATGGCCTCCA] | 4 | chr15 intronic | | *D15Ertd621e* | |
| GGGCAGGC[AGCTGGCCTCCA] | 4 | chr17 intronic | | *Lrfn2* | |
| GGTCAGTC[AGGTGGCCTCCA] | 4 | chr13 intergenic | | *NA* | |
| GGACATGC[ATATGGCCTCCA] | 4 | chr9 intergenic | | *Chst2* | |
| TTCCAGCC[TTATGGCCTCCA] | 4 | chr15 exonic | | *Oplah* | |
| GCACCTCC[AGGTGGCCTCCA] | 4 | chr13 intronic | | *Adcy2* | |
| GTGCAGGG[GGATGGCCTCCA] | 4 | chr13 intronic | | *Cdyl* | |
| GGACATCT[ACATGGCCTCCA] | 4 | chr5 intergenic | | *4930487D11Rik* | |
| GTACCACA[GGATGGCCTCCA] | 4 | chr7 intronic | | *Igf1r* | |
| GTAATTCC[AGCTGGCCTCCA] | 4 | chr18 intronic | | *Hars* | |
| GTAACTCC[AGCTGGCCTCCA] | 4 | chr8 intergenic | | *Brf2* | |
| ATACACCC[CAATGGCCTCCA] | 4 | chr3 intergenic | | *Isg20l2* | |
| GAGCAGAC[AGAGGGCCTCCA] | 4 | chr6 intronic | | *Ube2h* | |
| GTATATGC[AGGTGGCCTCCA] | 4 | chr14 intergenic | | *Gm23652* | |
| **Guide 2** | **CCCGTGGTGCACTCCCACCTGAA** | | | | |
| **target_seq** | **mismatches** | | **locus** | | **gene** |
| TTCAGGTG[GGAGTGCACCAC] | 0 | | chrX exonic | | *Rab39b* |
| TGGTGGGG[GGAGTGCACCAC] | 4 | | chr9 intronic | | *AB124611* |
| GGCAGGTG[GCAGTGCACCAC] | 3 | | chr4 intergenic | | *Rps20* |
| GTCAAGTG[AGAGTGCACCAC] | 3 | | chr11 intronic | | *Gm12153* |
| TGGACGTG[GCAGTGCACCAC] | 4 | | chr8 intronic | | *Pdgfrl* |
| CACAGGAG[GCAGTGCACCAC] | 4 | | chr11 intronic | | *Col23a1* |
| TGCTGGAG[GAAGTGCACCAC] | 4 | | chr1 intronic | | *Gm37382* |
| TTTTGGAG[GAAGTGCACCAC] | 4 | | chr17 intronic | | *Lhcgr* |
| ATCAGGGC[TGAGTGCACCAC] | 4 | | chr19 intergenic | | *Scd1* |
| TGCAGTTG[AAAGTGCACCAC] | 4 | | chr16 intergenic | | *Rps10-ps2* |
| TTCGTGTG[GCTGTGCACCAC] | 4 | | chr7 intergenic | | *Plekhf1* |
| TTCGTGTG[GCTGTGCACCAC] | 4 | | chr9 intergenic | | *Gm28087* |
| TTCGGATG[GCCGTGCACCAC] | 4 | | chr13 intergenic | | *Arhgef28* |
| TTCAGCAG[AGAGCGCACCAC] | 4 | | chr18 intronic | | *Nedd4l* |
| TTCAGCAC[GGAGTTCACCAC] | 4 | | chr7 exonic | | *Synm* |
| TGCAGGAG[GAAGTACACCAC] | 4 | | chr4 intergenic | | *NA* |
| TTCAGATA[GGTGGGCACCAC] | 4 | | chr7 intergenic | | *NA* |
| TACATGTG[GAAGTGAACCAC] | 4 | | chr11 intronic | | *Sgcd* |
| CACAGGTG[GGAGCACACCAC] | 4 | | chr2 intronic | | *Pcsk2* |
| TTATGGTG[GGTGTGGACCAC] | 4 | | chr6 intergenic | | *RP23-4F16.8* |

**Table S2** Genotyping primers

| **Primer** | **Sequence** |
| --- | --- |
| m*Rab39b*_F | 5’ –CTGTACCAGTTCCGGCTCAT–3’ |
| m*Rab39b*_R | 5’ –CTGTAGTAGGCGCGAGTGATG–3’ |
| m*Rab39a*_F | 5’ –GCCTGCTGGAGATTGAGC–3’ |
| m*Rab39a*_R | 5’ –TTTAACCCCTTCCCATCCAT–3’ |
| m*Gapdh*_F | 5’ –AACGGGAAGCCCATCACC–3’ |
| m*Gapdh*_R | 5’ –CAGCCTTGGCAGCACCAG–3’ |

**Table S3** Riboprobe primers

| **Primer** | **Sequence** |
| --- | --- |
| m*Rab39b* Probe1 Forward | 5’ –AGTCCTGCCAGGCCACTG–3’ |
| m*Rab39b* Probe1 Reverse | 5’ –TATGCAGCAGCCAGTTTCTC–3’ |
| m*Rab39b* Probe2 Forward | 5’ –CTGTACCAGTTCCGGCTCAT–3’ |
| m*Rab39b* Probe2 Reverse | 5’ –CTGTAGTAGGCGCGAGTGATG–3’ |
