# Supplementary figures and images for: Distribution of Parkinson’s disease associated RAB39B in mouse brain tissue
Source: Mol Brain. 2020 Mar 30;13:52. doi: 10.1186/s13041-020-00584-7 (PMC7106796; doi:10.1186/s13041-020-00584-7)

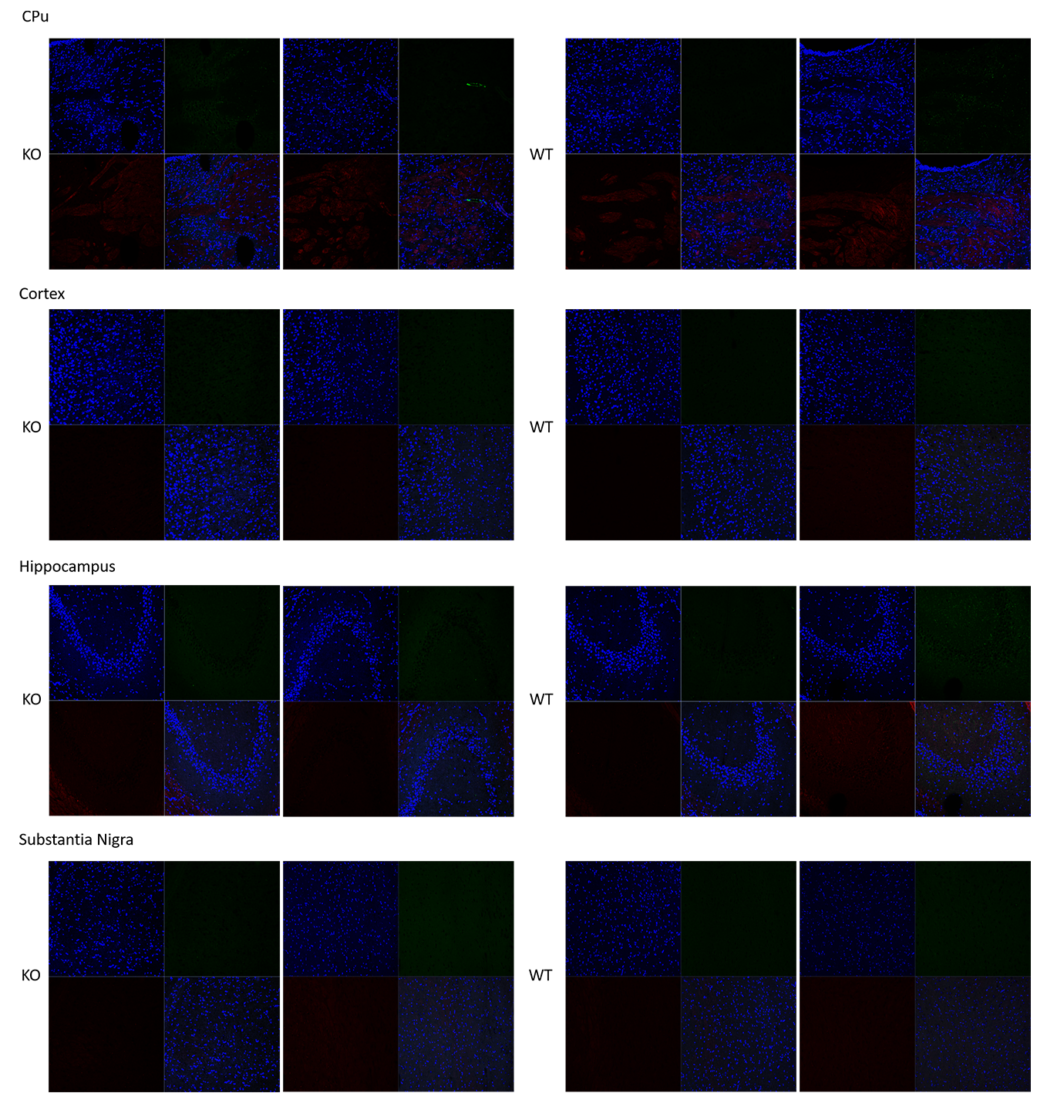

Supplement: Supplementary file 2 — Additional file 2: Figure 1. Control experiments for IHC. [file 13041_2020_584_MOESM2_ESM.tif]
